# Inhaled Liposomal Ciprofloxacin Protects against a Lethal Infection in a Murine Model of Pneumonic Plague

**DOI:** 10.3389/fmicb.2017.00091

**Published:** 2017-02-06

**Authors:** Karleigh A. Hamblin, Stuart J. Armstrong, Kay B. Barnes, Carwyn Davies, Thomas Laws, James D. Blanchard, Sarah V. Harding, Helen S. Atkins

**Affiliations:** ^1^CBR Division, Defence Science and Technology Laboratory, Porton DownSalisbury, UK; ^2^Aradigm Corporation, HaywardCA, USA; ^3^Biosciences, University of ExeterExeter, UK

**Keywords:** Plague, *Yersinia pestis*, liposomal drug delivery, ciprofloxacin, biological warfare agents, inhalation exposure

## Abstract

Inhalation of *Yersinia pestis* can lead to pneumonic plague, which without treatment is inevitably fatal. Two novel formulations of liposome-encapsulated ciprofloxacin, ‘ciprofloxacin for inhalation’ (CFI, Lipoquin^®^) and ‘dual release ciprofloxacin for inhalation’ (DRCFI, Pulmaquin^®^) containing CFI and ciprofloxacin solution, are in development. These were evaluated as potential therapies for infection with *Y. pestis*. In a murine model of pneumonic plague, human-like doses of aerosolized CFI, aerosolized DRCFI or intraperitoneal (i.p.) ciprofloxacin were administered at 24 h (representing prophylaxis) or 42 h (representing treatment) post-challenge. All three therapies provided a high level of protection when administered 24 h post-challenge. A single dose of CFI, but not DRCFI, significantly improved survival compared to a single dose of ciprofloxacin. Furthermore, single doses of CFI and DRCFI reduced bacterial burden in lungs and spleens to below the detectable limit at 60 h post-challenge. When therapy was delayed until 42 h post-challenge, a single dose of CFI or DRCFI offered minimal protection. However, single doses of CFI or DRCFI were able to significantly reduce the bacterial burden in the spleen compared to empty liposomes. A three-day treatment regimen of ciprofloxacin, CFI, or DRCFI resulted in high levels of protection (90–100% survival). This study suggests that CFI and DRCFI may be useful therapies for *Y. pestis* infection, both as prophylaxis and for the treatment of plague.

## Introduction

The Gram-negative bacterium *Yersinia pestis* is the causative agent of plague, a disease responsible for the death of an estimated 200 million people through devastating pandemics such as the Black Death (Perry and Fetherston, 1997). Due to improvements in living conditions and public health, outbreaks are now relatively uncommon. However, in the last 5 years there have been cases of plague reported in Uganda, Democratic Republic of Congo and Peru as well as sporadic disease occurring in the USA, China, and Mongolia (Organisation, 2016). Furthermore, plague is endemic in Madagascar where 308 people were infected and 81 died from the disease in the 2014–2015 season (Bertherat, 2015).

There are numerous cases throughout history of *Y. pestis* being used as a biological weapon (BW). In the Middle Ages, the bodies of dead plague victims were thrown over the walls of a besieged Caffa city by the Tartars (Wheelis, 2002). More recently, during the Second World War the Japanese offensive BW program is alleged to have dropped fleas infected with *Y. pestis* over parts of China (Harris, 1993). Even today there are concerns that plague could be used as a BW as *Y. pestis* could be accessed relatively easily (it can be found on every continent except Australia), there is the potential for person to person spread and the mortality rate of plague is high (Inglesby et al., 2000).

In natural outbreaks, *Y. pestis* infections have primarily been spread through bites from infected fleas, resulting in bubonic plague in humans (Perry and Fetherston, 1997). These patients suffer from swollen painful lymph nodes, known as buboes, as well as fever and physical exhaustion. Patients with positive blood cultures but no buboes are generally diagnosed with septicemic plague (Perry and Fetherston, 1997). However, it is inhalation of *Y. pestis* which results in the most severe form of the disease, pneumonic plague; if untreated this has a mortality rate of 100% (Crook and Tempest, 1992). Pneumonic plague usually begins with nondescript flu-like symptoms, which then quickly develop into severe pneumonia with coughing and bloody sputum. In the 2014–2015 outbreak in Madagascar, 7% of cases were pneumonic plague (Bertherat, 2015). Furthermore, pneumonic plague can develop secondarily following bubonic or septicemic plague and this also has a high fatality rate (Perry and Fetherston, 1997).

Currently, there is no licensed vaccine available for use in humans, although a plague vaccine has been developed and is in human phase II clinical trials (Hart et al., 2012). Current recommendations suggest treating plague with streptomycin or gentamicin (both of which require intravenous administration); potential alternatives are ciprofloxacin, doxycycline or chloramphenicol (Inglesby et al., 2000). In the event of a mass casualty situation, prophylaxis with oral doxycycline or ciprofloxacin is recommended (Inglesby et al., 2000) due to the ease of administration. However, to treat pneumonic plague, therapy must be initiated early as very high mortality rates have been observed in patients when therapy was not initiated within 24 h of symptoms developing (Inglesby et al., 2000).

Systemic antibiotics, in general, poorly treat lower respiratory infections, such as those associated with cystic fibrosis, and consequently inhaled antimicrobial agents which can directly target the lung have been developed (Zhou et al., 2015; Cipolla et al., 2016). A novel formulation of ciprofloxacin, utilizing liposome-encapsulation technology, has been developed by Aradigm Corporation specifically for inhalational delivery. Liposomal ‘ciprofloxacin for inhalation’ (CFI) can provide high, sustained concentrations of ciprofloxacin in the lung with once daily dosing (Bruinenberg et al., 2010). Furthermore, liposomes can be taken up by phagocytic cells enabling antibiotic delivery to the intracellular site of infection (Chono et al., 2008). A second formulation ‘dual release ciprofloxacin for inhalation’ (DRCFI), containing a mixture of free and liposome- encapsulated ciprofloxacin, has also been developed (Serisier et al., 2013). The free ciprofloxacin part of this formulation can immediately act upon the bacteria and may also act upon the host as ciprofloxacin has immunomodulatory activity (Dalhoff, 2005).

Both formulations have been evaluated in human clinical trials and DRCFI is currently in phase 3 clinical trials for *Pseudomonas aeruginosa* infection in patients with non-cystic fibrosis bronchiectasis (Bruinenberg et al., 2009, 2010; Serisier et al., 2013 and https://clinicaltrials.gov/ct2/show/NCT02104245). In addition to usefulness for public health indications these inhaled formulations may offer an advantage, compared to an oral or intravenous formulation, in the event of a deliberate release of an aerosolized BW agent. If administered early enough, inhalation of CFI/DRCFI could be used to target the initial site of infection (the lung following inhalation of aerosolized BW agents) and prevent establishment of infection. As there is considerable clinical data available, this product could be repurposed as a medical countermeasure for plague under the FDA animal rule following pivotal efficacy studies in animals. Therefore, to determine whether this formulation offers an advantage over therapies currently licensed for plague we have herein sought to compare its efficacy to ciprofloxacin administration in humans (approved routes including oral or intravenous).

In our previous studies, a single dose of aerosolized CFI administered at 24 h post-challenge has provided full protection against a lethal challenge of inhalational *Francisella tularensis* Schu S4 infection in a murine model (Hamblin et al., 2014a). In a non-lethal mouse model of *Coxiella burnetii* infection, intranasally instilled CFI administered at 24 h post-challenge prevented weight loss and the development of clinical signs associated with infection (Norville et al., 2014). However, the efficacy of neither CFI nor DRCFI has been evaluated in BW mouse models once the infection has disseminated.

*Yersinia pestis* infection in the Balb/c mouse model is well characterized with two distinct stages of infection (Heine et al., 2013, 2014). At first the infection is localized to the lung, and then it disseminates throughout the mouse resulting in high bacterial burdens in multiple organs. Therapy initiated during the first stage of infection models the administration of prophylaxis to patients before symptoms have appeared. Delaying the initiation of therapy until bacteria are disseminated enables an approximation of the efficacy of antibiotics in the treatment of symptomatic patients. Addressing the potential need for post-exposure prophylaxis and to treat symptomatic patients, in this study the Balb/c mouse has been used to evaluate the likely efficacy of CFI and DRCFI for both prophylaxis and treatment of pneumonic plague.

## Materials and Methods

### Bacteria

*Yersinia pestis* strain CO92 was cultured on blood agar base original (BAB) supplemented with 0.02% haemin or in BAB broth (15 g/L proteose peptone, 2.5 g/L liver digest, 5.0 g/L yeast extract, 5.0 g/L sodium chloride, all from Oxoid, UK). This strain has a minimum inhibitory concentration of <0.063 mg/L for ciprofloxacin (Russell et al., 1998). Bacteria were prepared for the animal challenge inoculum by incubating several colonies (grown for 48 h at 28°C on agar plates) in broth for 40 h at 28°C, with shaking at 180 rpm. Actual inoculum concentration was determined by enumeration of cultured bacteria on agar plates. All experiments with *Y. pestis* were carried out in a Class III microbiological safety cabinet complying with British Standard 5726.

### Animals

All experiments with mice were carried out in accordance with the UK Animal (Scientific Procedures) Act (1986). Female BALB/cAnNCrl (BALB/c) mice, 8–10 weeks old and 20 g (±4 g), were obtained from Charles River Laboratories (UK) where they were implanted with a sub-cutaneous Pico transponder (Uno BV, Netherlands), enabling individual animals to be traced throughout the study. Mice were caged in groups of 5 in polypropylene cages with a stainless steel mesh cover, integral water bottle holder and diet hopper in an ACDP (UK) level 3 rigid wall isolator, complying with British Standard 5726. Corn cob grade 10/14 (International Product Supplies [IPS], irradiated) was used as a nesting material and a range of environmental enrichment was used throughout the study including cellulose dome home and aspen wood wool (IPS). Husbandry conditions consisted of a 12-h light-dark cycle (350 to 400 Lux during the day, 10 Lux during the night), 19 to 23°C and 45 to 65% relative humidity. Irradiated Labdiet rodent diet 5002 (IPS) and water were freely available during the study. Mice were allowed to acclimatize to their home cage environment for five days before challenge.

### Antibiotics

Ciprofloxacin (Ciproxin^®^, Bayer), liposomal ciprofloxacin for inhalation (CFI) (Aradigm, USA) and dual release ciprofloxacin for inhalation (DRCFI) (Aradigm, USA) were used in this study. Ciproxin, 2 mg/ml solution for infusion, and CFI (ARD-3100, Lipoquin^®^), 50 mg/ml liposome-encapsulated ciprofloxacin (expressed as hydrochloride) were used as supplied. DRCFI (ARD-3150, Pulmaquin^®^), 35 mg/ml ciprofloxacin hydrochloride, was produced by mixing equal volumes of CFI and ciprofloxacin hydrochloride solution for inhalation (20 mg/ml) (Aradigm, USA) immediately prior to use.

### Identification of Appropriate Dosing Regimens

Mice were administered either ciprofloxacin, CFI, or DRCFI. Aerosolized CFI and DRCFI were administered using the inhalational therapy system (ITS) (Hamblin et al., 2014a), with 6 ml of the drug formulation placed in the nebulizer. Mice were exposed to the CFI (*n* = 30) or DRCFI (*n* = 30) aerosols for 20 or 15 min, respectively. Not all of drug in the nebulizer was aerosolized, for example following a 20 min exposure approximately 4 ml of CFI was aerosolized with 2 ml remaining in the nebulizer. The majority of the drug passed through the mouse exposure chamber and was collected in the impinger placed after the exposure chamber (the air is moving through the exposure chamber at 6 L/min whereas mouse respiration is 20 ml/min).

A single dose of ciprofloxacin (30 mg/kg) was administered to mice (*n* = 30) via the intraperitoneal (i.p.) route. The mice (*n* = 3 /time point/group) were culled at 1, 10, 20, or 30 min and 1, 1.5, 2, 4, 8, 12 h following ciprofloxacin administration and 1, 15, or 30 min and 1, 2, 4, 6, 10, 18, or 24 h following DRCFI or CFI administration. Blood sampling points were chosen based upon the short half-life of ciprofloxacin and longer half-life of CFI (Hamblin et al., 2014b). Blood and lungs (whole organ) were collected *post mortem* for analysis. The lung doses following CFI or DRCFI administration were calculated using the concentration of ciprofloxacin in the lung samples at 1 min post-administration.

The concentration-time profile of ciprofloxacin in blood and lung homogenate was determined using liquid chromatography mass spectrometry (LC-MS) as previously described (Hamblin et al., 2014a; Norville et al., 2014). Non-compartmental pharmacokinetic (PK) analysis of the mean concentration–time profiles of ciprofloxacin in the mouse lung and plasma was performed using WinNonlin v.6.1 (Certara, USA). One-compartmental PK analysis of the 15 min DRCFI exposure was also completed to enable simulation of the longer, 30 min, DRCFI exposure.

For comparison purposes, human lung doses were estimated from *in vitro* measurements of droplet size distribution and emitted dose from the jet nebulizer used clinically (Cipolla et al., 2010).

### Antibiotic Efficacy in BALB/c Mice

Two separate animal experiments were conducted; the first experiment determined the efficacy of antibiotics administered at 24 h post-challenge (the post-exposure prophylaxis study) and the second experiment determined the impact of delaying the initiation of therapy until 42 h post-challenge (the treatment study). In these studies, the *Y. pestis* aerosol was generated using a Collison nebulizer containing 20 ml of *Y. pestis* strain CO92 at a concentration of approximately 2 × 10^9^ CFU/ml and conditioned using a modified Henderson apparatus (Druett, 1969). Mice were exposed to the aerosol, in groups of 20, for 10 min via a head-only exposure chamber, with aerosol sampling of the aerosol chamber performed using an all-glass impinger (AGI-30; Ace Glass, USA) containing PBS. Each mouse received a retained dose of approximately 1 × 10^4^ CFU in the treatment study or 3 × 10^4^ CFU in the prophylaxis study (determined though enumeration of the bacterial concentration in the aerosol with a calculation (Harper and Morton, 1953) using Guyton’s formula (Guyton, 1947)). The aerosol exposure system has previously been validated for *Y. pestis* (Thomas et al., 2009). Exposing animals to an aerosol of 1.52 × 10^9^ CFU of *Y. pestis*, resulted in a lung dose of 1.76 × 10^4^ ± 0.085 × 10^4^ CFU (experimentally determined) compared to an estimated retained dose of 1.9 × 10^4^ CFU (Thomas et al., 2009).

To determine the bacterial burden of mice administered therapy as prophylaxis or treatment, groups of 5 mice received a single dose of i.p. ciprofloxacin, i.p. PBS, aerosolized empty liposomes, aerosolized CFI or aerosolized DRCFI (administered as described above, but with a 30 min exposure for DRCFI) at 24 or 42 h post-challenge. A 30 mg/kg dose of i.p. ciprofloxacin administered produces a drug exposure which is similar to the drug exposure observed in humans following a 500 mg dose of oral ciprofloxacin. The empty liposome control was administered using the ITS (Hamblin et al., 2014a) with 6 ml placed in the nebulizer and mice exposed to the aerosol for 20 min. Mice received approximately 2.8 mg/kg (lung dose) of lipid which matches the lipid lung dose of CFI and DRCFI (2.7 and 3.2 mg/kg). All mice were culled at the onset of clinical signs in the control group of mice administered PBS (at approximately 60 h post-challenge). Spleens and lungs were harvested and processed to determine bacterial load. The limit of detection of bacteria in these organs was 50 CFU/g in the lung and 200 CFU/g in the spleen.

To determine survival following the *Y. pestis* challenge, groups of 10 mice were administered therapy from 24 or 42 h post-challenge. The groups received a single dose of antibiotic or 3 days of therapy; the mice receiving 3 days of therapy were dosed with ciprofloxacin twice daily whereas CFI, DRCFI and empty liposome control were administered once daily to model the human dosing regimen (Serisier, 2012; Serisier et al., 2013). Mice were observed for 35 days post-challenge and clinical signs were recorded twice daily using a scoring system describing the extent of piloerection, hunching, eye problems and locomotion with scores of 0, 1, or 2 specified for each category. Humane end-points were used to identify moribund mice and minimize suffering.

### Statistical Analysis

Graphs were constructed with PRISM (v6.0, GraphPad), statistical analysis was conducted with SPSS (v21.0, IBM) and power calculations were conducted with SPSS SamplePower (v3.0 IBM). In previous *Y. pestis* studies using similar doses of bacteria, mice began to succumb approximately 2.5 days post infection with a high hazard rate (probability that each mouse will succumb on a particular day) of approximately 1 (Thomas et al., 2009). We estimated that groups of 10 mice would be sufficient to provide a power of ∼80% with reduction in hazard rate to 0.25 (i.e., 25% of the normal virulence). No equivalent CFI/DRCFI treatment survival data was available for power calculation. For assessing differences in bacterial load, we estimated that the group size of 5 would be sufficient to show differences of 1.5 log10s with a ∼80% power as previous data had suggested that the standard deviation of *Y. pestis* colonization data (transformed to the logarithm of 10) would be around 0.5 (Thomas et al., 2009). The high frequency of animals where no bacteria could be recovered negated the possibility for using parametric analysis and Mood’s median tests with multiple comparisons used to compare bacterial colonization of groups. Where no bacteria could be isolated a number of 0 was assigned. Survival data were compared using Logrank tests. Differences of *p* < 0.05 were considered statistically significant.

## Results

### Identification of Appropriate Dosing Regimens of i.p. Ciprofloxacin and Inhaled CFI or DRCFI from Human PK

The AUC/MIC (area under the curve/minimum inhibitory concentration) measurement has been shown to be the PK parameter most strongly associated with the efficacy of ciprofloxacin in animal models (Craig, 1998), although this has not been validated for local treatment of lung infections. In this study, administration of a single 30 mg/kg dose of ciprofloxacin, via the intraperitoneal route, to Balb/c mice resulted in a drug exposure which is similar to the drug exposure observed in humans following a 500 mg dose of oral ciprofloxacin (see AUC in **Table 1**).

**Table 1:** Pharmacokinetic parameters of ciprofloxacin in Balb/c mouse plasma and lung homogenate following a single dose of intraperitoneal ciprofloxacin, aerosolized CFI, or aerosolized DRCFI and comparison to clinical doses.

Drug	Species	Total administered Dose	Lung dose^∗^	Tissue	Cmax (μg/ml or μg/g)	Tmax (h)	AUC (μghr/ml or μghr/g)	T 1/2 (h)	CL (ml/h/kg or mg/h/kg)
Ciprofloxacin	Mouse (Balb/c)	30 mg/kg (i.p.)		Lung	76.6	0.02	35.0	1.4	855
				Plasma	16.9	0	11.6	1.1	2,593
	Human	500 mg (p.o.) (Lettieri et al., 1992)		Serum	2.7 ± 0.8	–	10.7 ± 2.6	5.7 ± 1.2	–
CFI	Mouse (Balb/c)	10 mg/kg^∗^	1 mg/kg (20 min exposure)	Lung^∗∗^	116	0.02	773	7.4	1.19
				Plasma	0.30	0.02	0.94	7.0	1,007
	Human	300 mg (initial loaded dose in nebulizer) (8)		Plasma	0.11	1.5	1.22 AUC(0-∞)	11.04	NA
DRCFI	Mouse (Balb/c)	4 mg/kg^∗^	0.4 mg/kg (15 min exposure)	Lung	54	0.5	356	4.8	1.08
				Plasma	0.32	0.25	0.80	11.3	424
		8 mg/kg^∗^	0.8 mg/kg (30 min exposure)	Lung	100.8^#^	0.02^#^	710^#^	4.9^#^	1.13^#^
				Plasma	0.40^#^	0.06^#^	1.76^#^	3.0^#^	454^#^
	Human	111 mg (initial loaded dose in nebulizer)^∗∗∗^		Plasma	0.17	0.33	0.92 AUC(0-∞)	9.4	NA

*Human data is shown for comparison, with overall exposure in the plasma (AUC) similar in the human clinical trials and this study, indicating that the mouse dose is a realistic surrogate for the human dose.*

*NA, not available*

*^∗^Lung dose (determined during pharmacokinetics study from 1 min time point). Extrapolation from Raabe et al. (1988) suggests that the 2.2 μm mass median diameter particles produced by the Pari LC Sprint Star results in 10% of the total inhaled dose depositions in the pulmonary, bronchial and trachea of mice, e.g., for a 1 mg/kg lung dose the total dose will be 10 mg/kg.*

*^#^ Predicted from 15 min exposure data using compartmental analysis performed by performed using WinNonlin*

*^∗∗^Data from Hamblin et al. (2014a) included for comparison.*

*^∗∗∗^P., Bruinenberg, D. Serisier, J. Blanchard, D. Cipolla, and I. Gonda. Effects and modulation of release rate of inhaled ciprofloxacin with liposomal formulations in healthy subjects and patients with bronchiectasis. Presented at European Respiratory Society Annual Congress, Barcelona, Spain. Abstract 5574, 2010. Dose used in Phase 2 and 3 clinical trials has been increased to 210 mg loaded dose to give a lung dose of 0.8 mg/kg (Aradigm, unpublished data). PK data for this dose will be available when the Phase 3 trial results are published.*

Inhalational drug scaling between humans and animals relies upon attempting to deliver the same amount of drug to the lung per kg of body weight. In Phase II human clinical trials with DRCFI, 6 ml (210 mg) was nebulized, the corresponding dose delivered to the lung is approximately 0.8 mg/kg, assuming a 50-kg patient (Aradigm Corporation, unpublished data) which is similar to the dose of CFI delivered to mice (see **Table 1**). In this study, exposure to DRCFI resulted in a lower inhaled ciprofloxacin dose (see **Table 1**). Therefore, in order to obtain a similar dose of CFI and DRCFI, the length of exposure to DRCFI was increased. **Table 1** shows the PK parameters of the shorter DRCFI exposure and the PK of the longer exposure simulated by the one-compartment models.

**FIGURE 1:**
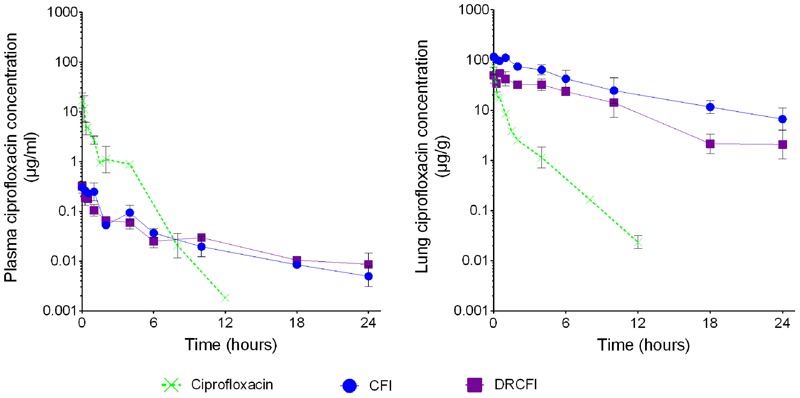
**Lung and plasma concentration-time profile of ciprofloxacin administered as intraperitoneal (IP) ciprofloxacin or aerosolized CFI or DRCFI to BALB/c mice.** Mice were dosed with 30 mg/kg of IP ciprofloxacin, 1 mg/kg lung dose of aerosolized CFI or 1 mg/kg lung dose of aerosolized DRCFI. Note the prolonged elimination of ciprofloxacin in lung and plasma as well as the reduced plasma concentrations when CFI or DRCFI is administered by inhalation directly to the lung.

The PK parameters of CFI and DRCFI demonstrate the different distributions of ciprofloxacin following administration of these therapies compared to ciprofloxacin administered by the intraperitoneal route. As previously demonstrated in humans (Bruinenberg et al., 2009, 2010; Serisier et al., 2013), in the Balb/c mouse administration of CFI or DRCFI via the inhalational route results in low systemic concentrations of ciprofloxacin compared to ciprofloxacin delivered by the intraperitoneal route (see **Figure 1**) with a greater than 10-fold decrease in the plasma total exposure (as demonstrated by the AUC). Conversely, and as may be expected, inhalational administration of CFI or DRCFI produces a higher C_max_ and extended half-life in the lung (see **Figure 1**) resulting in a 20-fold higher overall exposure in the mouse lung compared to ciprofloxacin delivered by the intraperitoneal route.

### A Single Dose of CFI Administered at 24 h Post-Challenge Offers a Higher Level of Protection Against *Y. pestis* than Ciprofloxacin

**FIGURE 2:**
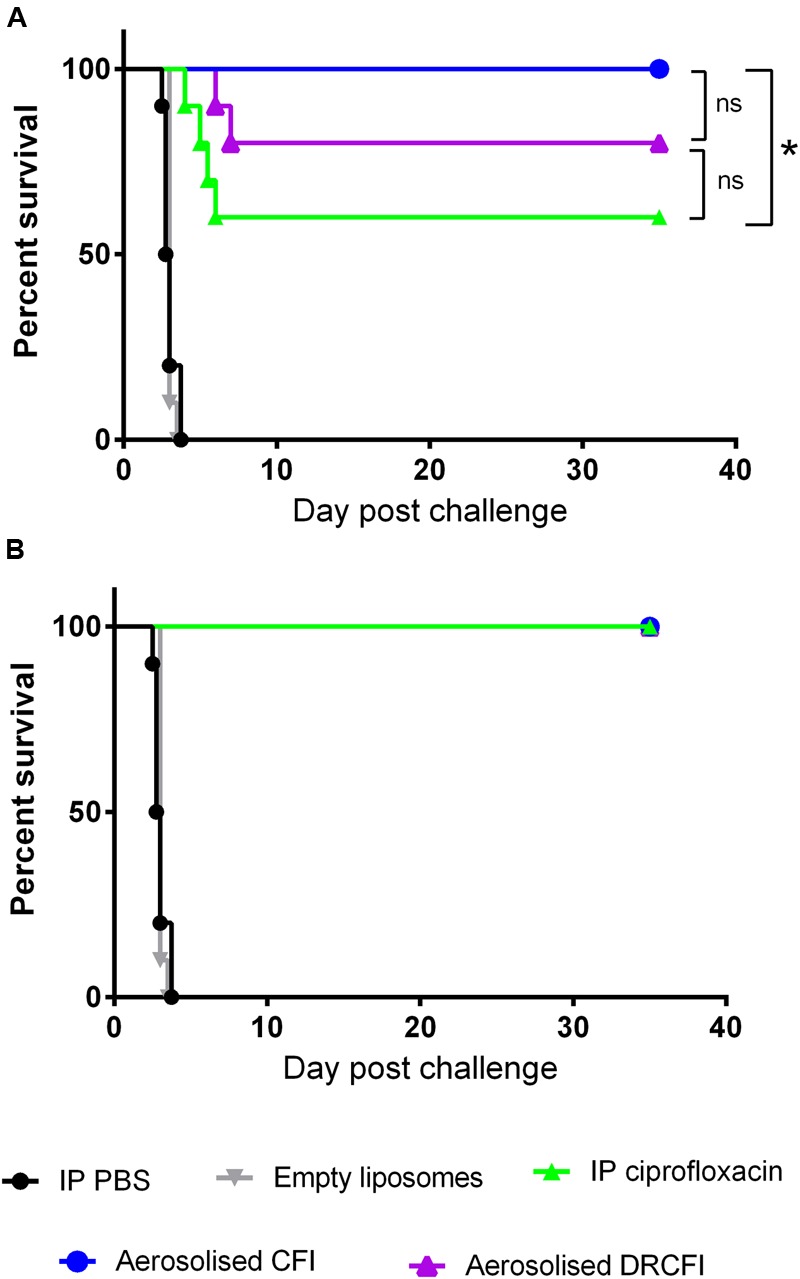
**Therapeutic efficacy of intraperitoneally delivered ciprofloxacin, aerosolized CFI or DRCFI prophylaxis in a mouse model of inhalational *Yersinia pestis* infection.** BALB/c mice (*n* = 10/group) were challenged via the aerosol route with approximately 1 × 10^4^ CFU *Y. pestis* strain CO92 and treated 24 h post-challenge with 30 mg/kg of intraperitoneal (IP) ciprofloxacin, 300 μl IP PBS, aerosolized empty liposomes (2.8 mg/kg lipid), 1 mg/kg lung dose of aerosolized CFI or DRCFI (2.7 and 3.2 mg/kg lipid, respectively). Graphs show the survival of mice following **(A)** 1 dose of therapy and **(B)** 3 days of therapy. All treatments improved survival compared to PBS or empty liposome treatment (*P* < 0.001). Asterisks indicate other significant differences in survival, ^∗^ for *p* = 0.029, ns indicates no significant difference (*P* > 0.05). Note: animals receiving PBS, ciprofloxacin, CFI, and DRCFI were exposed at the same time to the same aerosol.

First, an early initiation of therapy was investigated, with therapy administered from 24 h post-challenge. Using the Balb/c mouse model of aerosolized *Y. pestis* infection, bacteria could be detected (∼2 × 10^3^ CFU/ml) in the lung of all animals at this time, but no bacteria could be detected in the spleen or blood. The control mice (treated with PBS or empty liposomes) succumbed to the infection by day 4 post-challenge whereas a single dose of ciprofloxacin resulted in 60% of mice surviving the lethal challenge. However, a single dose of CFI afforded significantly better protection than ciprofloxacin with all mice surviving until the end of the experiment at day 35 (*P* = 0.029). There was no significant difference between DRCFI and ciprofloxacin or CFI, with a single dose of DRCFI treatment resulted in 80% survival. When therapy was administered for 3 days, all therapies provided full protection (see **Figure 2**).

### A Single Dose of CFI or DRCFI Administered at 24 h Post-Challenge Reduces Bacterial Burden in Lung and Spleen to Below the Limit of Detection at 60 h Post-Challenge

**FIGURE 3:**
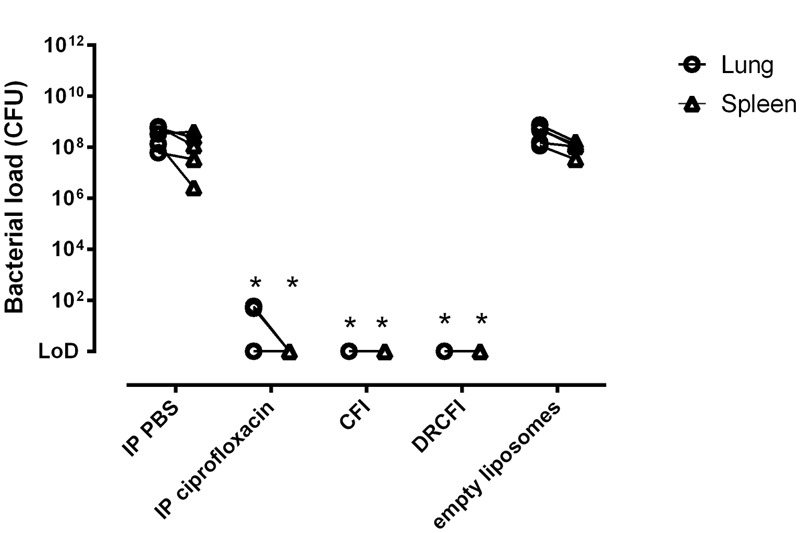
**The effect of ciprofloxacin, CFI or DRCFI prophylaxis on bacterial burden in a mouse model of inhalational *Y. pestis* infection.** BALB/c mice (*n* = 5/group) were challenged via the aerosol route with approximately 1 × 10^4^ CFU *Y. pestis* CO92 and treated 24 h post-challenge with a single dose of 30 mg/kg intraperitoneal (IP) ciprofloxacin, 300 μl IP PBS, aerosolized empty liposomes (2.8 mg/kg lipid), 1 mg/kg lung dose of aerosolized CFI or DRCFI (2.7 and 3.2 mg/kg lipid, respectively). Graphs show bacterial burden in lung and spleen at onset of clinical signs in control groups (approximately 2.5 days post-challenge). Lines connect spleen and lung loads from the same animal. LOD indicates samples which were below the limit of detection. Asterisks indicate significant differences in bacterial burden compared to the relevant control (i.e., CFI compared to empty liposomes, IP ciprofloxacin compared to IP PBS), ^∗^for *P* < 0.05.

To further differentiate between CFI, DRCFI, and ciprofloxacin, the bacterial burden in lungs and spleens of infected mice was determined at the onset of clinical signs in the control animals (those treated with PBS or empty liposomes). The bacterial load was high in both lungs and spleens in the control groups whereas all three antibiotics had profound effects on bacterial load (see **Figure 3**). No bacteria were isolated from the spleen of any antibiotic treated animal and, when compared to controls, this represents significant reductions in spleen bacterial loads (*P* = 0.023). CFI and DRCFI treatment also reduced the lung bacterial loads to lower than the limit of detection, significantly different to the empty liposome control (*P =* 0.023). Intraperitoneal ciprofloxacin did reduce the lung bacterial load compared to controls treated with intraperitoneal PBS (*P* = 0.023) (see **Figure 3**), however, bacteria could still be detected in 2 out of 5 mouse lungs. There were no significant differences in bacterial loads among the different therapies.

### CFI and DRCFI Can Offer a High Level of Protection against *Y. pestis* When Therapy is Delayed

**FIGURE 4:**
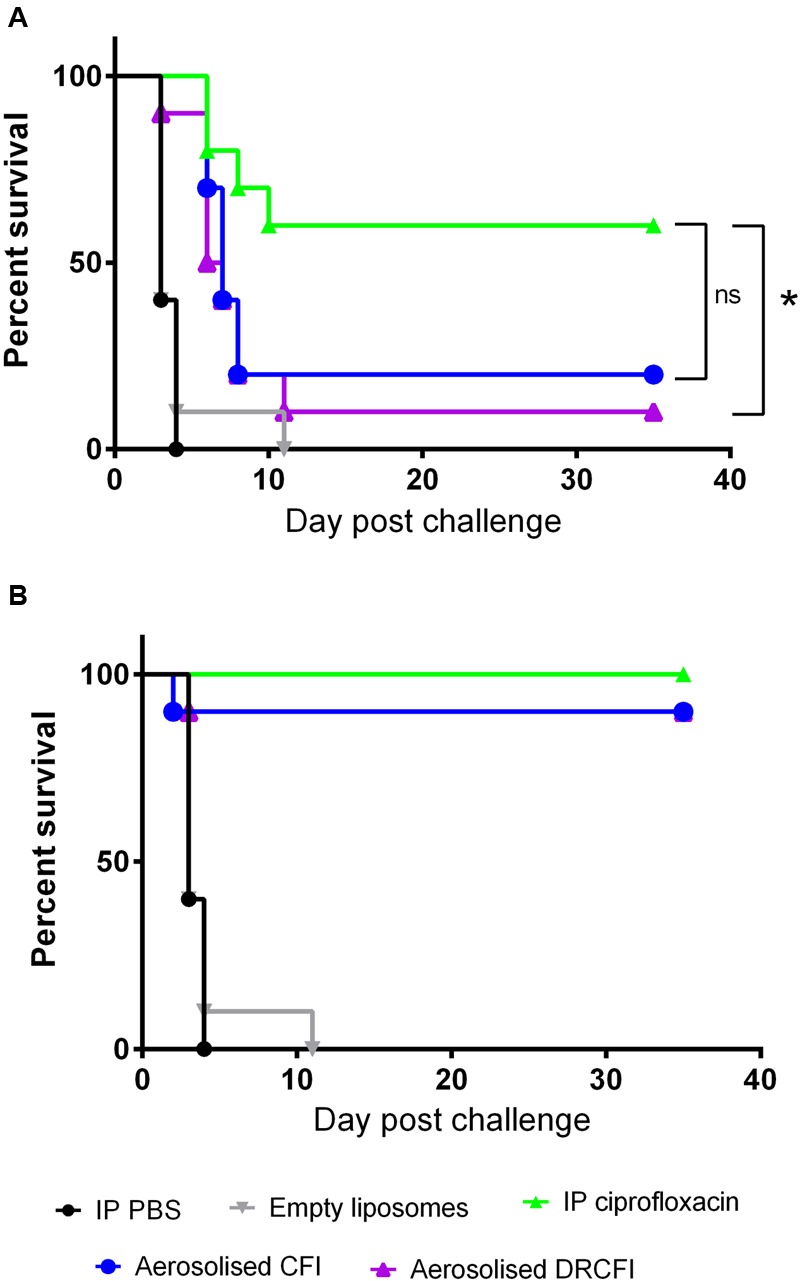
**Therapeutic efficacy of ciprofloxacin, CFI or DRCFI treatment in a mouse model of inhalational *Y. pestis* infection.** BALB/c mice (*n* = 10/group) were challenged via the aerosol route with approximately 3 × 10^4^ CFU *Y. pestis* CO92 and treated 42 h post-challenge with 30 mg/kg of intraperitoneal (IP) ciprofloxacin, 300 μl IP PBS, aerosolized empty liposomes (2.8 mg/kg lipid), 1 mg/kg lung dose of aerosolized CFI or DRCFI (2.7 and 3.2 mg/kg lipid, respectively). Graphs show the survival of mice following **(A)** 1 dose of therapy and **(B)** 3 days of therapy. All treatments improved survival compared to PBS or empty liposome treatment (*P* < 0.001). Asterisks indicate significant differences in survival, ^∗^ for *p* = 0.016, ns indicates there were no significant differences (*P* < 0.05).

The efficacy of CFI and DRCFI against systemic *Y. pestis* infection was investigated. Specifically, administration of therapy was delayed until 42 h. At this time point, there was a high bacterial burden in the lungs (∼1 × 10^6^ CFU/organ) and bacteria could be detected in the blood and spleen of 3 out of 5 mice with an average bacterial burden of ∼3 × 10^4^ CFU/ml and ∼3 × 10^5^ CFU/organ, respectively. All control mice succumbed to infection by day 4 except for 1 mouse dosed with empty liposomes which survived until day 11 post-challenge. A single dose of all three therapies did increase time to death compared to the controls (*P* < 0.001) but did not offer a high level of protection. A single dose of ciprofloxacin resulted in 60% survival, and a single dose of CFI or DRCFI resulted in 20 and 10% survival, respectively. Here, ciprofloxacin offered significantly better protection than DRCFI (*P* = 0.016) but not when compared to CFI (*P* = 0.060) (see **Figure 4**). However, impressively, three days of antibiotic therapy offered high levels of protection with 100, 90, and 90% survival afforded by ciprofloxacin, CFI, and DRCFI, respectively (see **Figure 4**).

### CFI Reduces Bacterial Burden in Lung and Spleen to Below the Detectable Limit at 60 h Post-challenge When Initiation of Therapy Is Delayed Until 42 h Post-challenge

**FIGURE 5:**
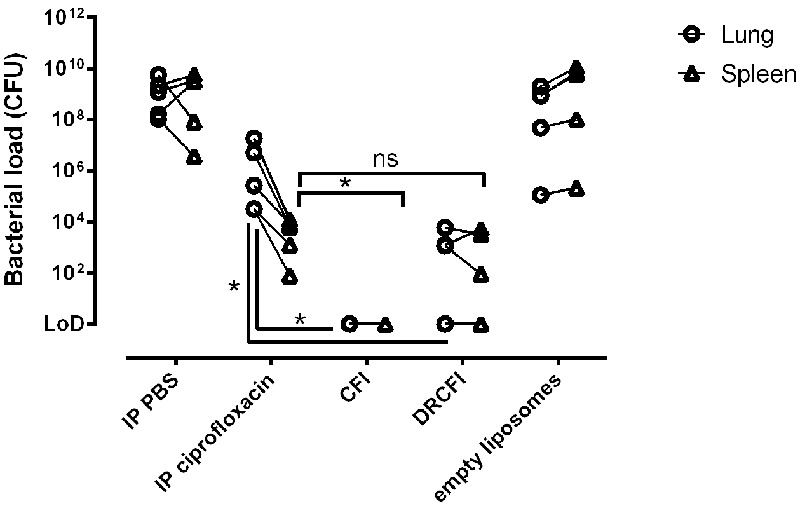
**Efficacy of ciprofloxacin, CFI or DRCFI treatment upon bacterial burden in a mouse model of inhalational *Y. pestis* infection.** BALB/c mice (*n* = 5/group) were challenged via the aerosol route with approximately 3 × 10^4^ CFU *Y. pestis* CO92 and treated 42 h post-challenge with a single dose of 30 mg/kg intraperitoneal (IP) ciprofloxacin, 300 μl IP PBS, aerosolized empty liposomes (2.8 mg/kg lipid), 1 mg/kg lung dose of aerosolized CFI or DRCFI (2.7 and 3.2 mg/kg lipid, respectively). Graphs show bacterial burden in lung and spleen at onset of clinical signs in control groups (approximately 2.5 days post-challenge). Lines connect spleen and lung loads from the same animal. LOD indicates samples which were below the limit of detection. All therapies significantly reduced bacterial burden compared to their relevant control (*P* < 0.05). Asterisks indicate significant differences in bacterial burden between therapies, ^∗^for *P* < 0.05 and ns for not significant.

The bacterial burden at the onset of clinical signs in control animals challenged with *Y. pestis* was examined when therapy was delayed until 42 h post-challenge. High bacterial loads were present in the lungs and spleens of the control animals. In comparison, bacterial numbers were reduced to levels below a measureable threshold in the lungs and spleen of all the mice treated with CFI and 3 of the 5 mice treated with DRCFI. A comparison of bacterial numbers in the spleen and lung indicated that the bacterial load had been reduced by all three therapies when compared to their relevant control groups (*P* = 0.023). Furthermore, both CFI and DRCFI treatment significantly reduced bacterial burden in the lung compared to ciprofloxacin treated mice (*P* = 0.023). CFI treatment also significantly reduced the spleen bacterial burden compared to ciprofloxacin treatment (*P* = 0.023) (see **Figure 5**). However, there was no significant difference in spleen bacterial burden following DRCFI or ciprofloxacin therapy. In addition, there was no significant difference in the lung or spleen bacterial burden of CFI and DRCFI treated mice.

## Discussion

In the event of a deliberate release or a natural outbreak of pneumonic plague, large numbers of patients and contacts may have to receive antibiotics. Due to the low numbers of natural cases, animal models are needed to evaluate the efficacy of antibiotics against pneumonic plague. The well-characterized Balb/c mouse model of pneumonic plague enables antibiotic evaluation for prophylaxis and, by delaying therapy, for treatment. In reality, those requiring treatment are likely to be in-patients in a hospital, with dedicated medical care and the potential for multiple therapies administered by medical staff. In contrast, those requiring prophylaxis are likely to have different requirements such as a therapy which can be taken without medical supervision and with very few side effects. Those receiving prophylaxis may include doctors or soldiers who need to remain active and able to perform their duties.

Delivery of a potent antibiotic directly to the site of the primary infection, the lung, provides the opportunity for much higher antibiotic concentration in proximity of the infection and much lower systemic exposure to avoid systemic side-effects. Indeed, inhaled CFI has been shown to be safe and well tolerated in a number of patient populations (Bruinenberg et al., 2009, 2010; Serisier et al., 2013). In addition, due to the altered route of administration inhaled CFI or DRCFI therapy should minimize gastro enteric side effects compared to oral ciprofloxacin. The encapsulation of ciprofloxacin in liposomes enables the antibiotic to be effective when delivered by inhalation. Free ciprofloxacin delivered by the inhalation route has a very short half-life (approximately 1 h) and is not an effective antimicrobial (Conley et al., 1997; Wong et al., 2003). In addition, the free ciprofloxacin aerosol is very irritating to the lung and is not tolerated very well (Aradigm, unpublished). For these reasons an inhaled free ciprofloxacin formulation has not been developed for clinical use.

In this study, when CFI or DRCFI were administered as prophylaxis, at 24 h post challenge, they appear to be as good as, i.p., ciprofloxacin at decreasing the bacterial burden in the lung and spleen and thus reducing the lethality of plague. This may be explained by the PK data which demonstrates that administration of CFI or DRCFI greatly increases the overall exposure to ciprofloxacin in the lung compared to ciprofloxacin administered by intraperitoneal injection. This high concentration of ciprofloxacin could prevent the disease from disseminating from the initial site of infection in the lung.

Delaying CFI or DRCFI treatment to 42 h post-challenge, a time point at which the infection has become systemic, also provided high levels of protection. This was somewhat unexpected as the systemic exposure to ciprofloxacin (measured by AUC in plasma) is more than 10-fold lower than ciprofloxacin delivered by the intraperitoneal route. Furthermore, CFI and DRCFI were able to reduce bacterial load in the spleen. This may be explained by the hypothesis that liposome-encapsulated antibiotics, like CFI, are taken up by macrophages in a similar manner to the phagocytosis of bacteria (Alving, 1988; Chono et al., 2008) and can alter the behavior of macrophages by increasing phagocytosis and nitric oxide production (Wong et al., 2000). If this is the case, ciprofloxacin loaded macrophages may translocate from the lung to other organs around the body (including the spleen) enabling a reduction in bacterial burden far from the lung. However, although CFI treatment appears to reduce bacterial burden to a greater extent than ciprofloxacin, this was not reflected in the pattern of survival, where a single dose of CFI resulted in only a low level of protection. As the disease progresses *Y. pestis* moves from an intracellular to extracellular pathogen, therefore although there are low bacterial numbers in the site of treatment (lung) and the leukocyte rich tissues (spleen) due to CFI treatment there may be other foci of infection enabling the disease to re-emerge. However, increasing the therapy regimen of CFI or DRCFI to three days enables the systemic infection to be successfully treated and offers a high level of protection.

In human clinical trials treating *Pseudomonas aeruginosa* infections in patients with non-cystic fibrosis bronchiectasis, DRCFI appeared more efficacious than CFI in terms of reduction of bacterial burden in the sputum (Serisier, 2012; Serisier et al., 2013). The free ciprofloxacin component has been hypothesized to provide an immediately acting antibiotic, while the liposome-encapsulated component allows the concentration of ciprofloxacin to remain high, through the slow release of ciprofloxacin from the liposomes. In the *Y. pestis* mouse infection model used in this study, there appeared to be no significant difference between the formulations. Perhaps this is due to differences in the pathogenesis of *P. aeruginosa* in bronchiectasis and a *Y. pestis* infection. Alternatively, this may be a limitation of the mouse model, with differences in the efficacy of CFI and DRCFI not reflective of the situation in humans. Further work investigating efficacy in alternative models of infection, such as NHPs, is required to determine whether CFI or DRCFI may be more effective against *Y. pestis.*

To summarize, in this study liposome-encapsulated antibiotics delivered by the inhalational route offer significant protection against *Y. pestis* when administered as post-exposure prophylaxis or as treatment. These results combined with previously published data showing the efficacy of CFI against *F. tularensis* and *C. burnetii* suggest that CFI and DRCFI have broad spectrum activity and the potential for use against multiple inhaled bacterial pathogens that pose a BW threat. Further work with CFI and DRCFI is therefore warranted, with the view to make a readily available, self-administrable, effective and safe broad-spectrum product for prophylaxis and treatment even though the exact nature of the pathogen may not be known.

## Ethics Statement

The animal studies reported in this paper were carried out in accordance with the UK Animal (Scientific Procedures) Act (1986).

## Author Contributions

KH, SH, JB, SA, and HA conceived the concept and designed the experiment. KH, KB, and CD conducted the experiments. KH, SA, and TL performed the data analysis. KH, HA, and JB wrote the manuscript. All the authors reviewed the draft and approved this manuscript for publication.

## Conflict of Interest Statement

JB is an employee and stockholder of Aradigm Corp. and the other authors declare that the research was conducted in the absence of any commercial or financial relationships that could be construed as a potential conflict of interest.
